# False-positive Malaria Rapid Diagnostic Tests are Prevalent Among Children Under 5 Years of Age in Uganda

**DOI:** 10.1093/infdis/jiaf604

**Published:** 2025-11-28

**Authors:** Caitlin A Cassidy, Bonnie E Shook-Sa, Ross M Boyce, Emily J Ciccone, Emily W Gower, Amber M Young, Jessie K Edwards

**Affiliations:** Department of Epidemiology, Gillings School of Global Public Health, University of North Carolina at Chapel Hill, Chapel Hill, North Carolina, USA; Nuffield Department of Population Health, University of Oxford, Oxford, United Kingdom; Department of Biostatistics, Gillings School of Global Public Health, University of North Carolina at Chapel Hill, Chapel Hill, North Carolina, USA; Department of Epidemiology, Gillings School of Global Public Health, University of North Carolina at Chapel Hill, Chapel Hill, North Carolina, USA; Institute for Global Health and Infectious Diseases, University of North Carolina at Chapel Hill, Chapel Hill, North Carolina, USA; Carolina Population Center, University of North Carolina at Chapel Hill, Chapel Hill, North Carolina, USA; Institute for Global Health and Infectious Diseases, University of North Carolina at Chapel Hill, Chapel Hill, North Carolina, USA; Department of Epidemiology, Gillings School of Global Public Health, University of North Carolina at Chapel Hill, Chapel Hill, North Carolina, USA; Department of Biostatistics, Gillings School of Global Public Health, University of North Carolina at Chapel Hill, Chapel Hill, North Carolina, USA; Department of Epidemiology, Gillings School of Global Public Health, University of North Carolina at Chapel Hill, Chapel Hill, North Carolina, USA; Carolina Population Center, University of North Carolina at Chapel Hill, Chapel Hill, North Carolina, USA

**Keywords:** Malaria, rapid diagnostic tests, antimicrobial resistance, false-positive, children under 5

## Abstract

**Background:**

Malaria rapid diagnostic tests (mRDTs) are a cornerstone of malaria testing and treatment efforts globally. However, positive mRDT results can occur after treatment due to antigen persistence, even in the absence of malaria parasites. False-negative mRDTs are well-described, but less is known about the prevalence and consequences of such false-positive results.

**Methods:**

We estimated the prevalence of false-positive mRDTs, defined as mRDT(+)/microscopy(−), using data from the 2018–2019 Uganda Malaria Indicator Survey (MIS). Children aged <5 years (under-5s) with paired mRDT and microscopy results were included. We estimated the prevalence of false-positive mRDTs among microscopy(−) children using survey weights. We fit bivariate generalized linear models to estimate the prevalence difference (PD) of false-positive mRDTs for pre-specified covariates. We constructed cross-validated weighted lasso regression models to determine which variables best predict false-positive mRDTs among children with recent fever.

**Results:**

The prevalence of false-positive mRDTs was 10.7% (849/6786) and was strongly correlated with region-level transmission intensity. Prevalence was higher among children with recent fever (PD: 17.2%; 95% CI: 13.7%, 20.6%), recent antimalarial use (14.7%; 7.1%, 22.3%), and comorbid anemia (8.1%; 5.9%, 10.3%). Prevalence was lower among those with recent antibiotic use (−17.6%; −22.5%, −12.7%). A model with clinical, environmental, and household variables better predicted false-positive mRDTs (weighted AUC = 0.79) than individual models.

**Conclusions:**

False-positive mRDTs are prevalent among under-5s in the 2018–19 Uganda MIS and lead to overestimates of community-level malaria prevalence. These results suggest that false-positive mRDTs may also contribute to misdiagnosis and unnecessary antimalarial use in clinical settings.

## Teaser

In the 2018–2019 Uganda Malaria Indicator Survey, false-positive mRDTs were frequent among children under 5. Clinical factors can be used to predict false-positive results among children with recent fever. Antimalarial overprescription due to false-positive mRDTs may accelerate emerging drug resistance.

The introduction of malaria rapid diagnostic tests (mRDTs) has contributed to increased testing for malaria [1]. mRDTs are fast and inexpensive, though less accurate than expert microscopy [2], which is more resource intense and requires skilled personnel. In Uganda, despite being the gold standard, microscopy is infeasible in most care-seeking settings. Malaria rapid diagnostic tests, however, are widely used in point-of-care malaria management, and can be accessed at drug shops, formal health clinics, and visits with community health workers [2]. They detect malarial antigen in an individual's blood, most commonly histidine rich protein II (HRP-2) [3].

While misclassification due to false-negative mRDTs has been well-described, less is known about the impact of false-positive results [4]. The HRP-2 antigen remains in the bloodstream and is detectable by mRDT even after treatment of malaria infection and clearance of the parasite, which can produce false-positive results. Estimates of the mean half-life of HRP-2 range from 3 to 9 days [5–7], but variability in the length of mRDT positivity has been demonstrated, up to 63 days after malaria treatment and the clearance of parasites [8]. Children tend to remain mRDT-positive longer than adults [8], and higher parasitemia at time of initial testing is associated with longer antigen persistence [9, 10]. In addition, in one study, the median length of mRDT positivity was longer in a high transmission area compared to a low transmission area [9]. Beyond age, transmission intensity, and parasitemia, little is known about other correlates of false-positive mRDT results.

False-positive mRDTs could pose serious consequences for management of nonmalarial febrile illnesses across sub-Saharan Africa (SSA). Nonmalarial causes of fever may go untreated in the presence of false-positive mRDTs. Further, progress toward malaria elimination has been hampered in part by the increasing resistance of *Plasmodium* species to antimalarial drugs. Parasite mutations are associated with partial artemisinin resistance, which makes antimalarial drugs, including artemisinin-based combination therapies (ACTs), the preferred treatment, less effective [11]. Uganda may be especially at risk for resistance to ACTs, due to the having one of the highest transmission burdens in SSA [11], and because mutations in *P. falciparum* have emerged in recent years [12]. Therefore, stewardship of ACTs is crucial to malaria control efforts.

The overarching goal of this study is to describe the epidemiology of false-positive mRDTs among children under 5 years of age (under-5s) in Uganda. To achieve this, we describe the prevalence and spatial variation of false-positive mRDTs among microscopy-negative children in Uganda, both nationally and by region, using the 2018–2019 Uganda Malaria Indicator Survey (UMIS). We estimate bivariate associations between false-positive mRDTs and demographic, clinical, and environmental factors. In addition, we develop prediction models to identify factors among under-5s with recent fever who are more likely to have a false-positive mRDT.

## METHODS

### Data Source and Population

We analyzed cross-sectional data from the most recent UMIS, conducted from December 2018 to January 2019. The UMIS is a nationally representative complex survey designed to estimate national and regional malaria prevalence among under-5s. All children aged 0–59 months in sampled households were eligible for biomarker testing, including microscopy and mRDT for malaria, and hemoglobin (Hb) for anemia.

We included all children aged 0–59 months who underwent paired malaria testing via mRDT and microscopy, had valid results (eg, positive or negative), and slept in the household in which they were tested the night prior to the survey. Children with missing mRDT or microscopy results were excluded. Trained field workers performed HRP-2 mRDTs (SD BIOLINE Malaria Ag *P.f*) and collected blood smears for microscopy. Child demographic and clinical characteristics and maternal demographic characteristics were reported by the mother via the women's questionnaire. Household demographic characteristics assessed by the household questionnaire were reported by any household member(s) aged 15 and older during the household interview.

### Predictor Variables

Candidate predictors were selected a priori if they were hypothesized to be associated with malaria transmission or false-positive mRDTs. Child demographic variables included the child's age (reported in months), sex, and whether they slept under a long-lasting insecticidal net (LLIN) the night prior to the survey. Clinical variables included anemia (Hb <11 g/dL), moderate anemia, (Hb < 8 g/dL), and whether in the 2 weeks prior to the survey the child had a fever, sought care for a fever, received antimalarials, antibiotics, antipyretics, or had blood taken from the finger or heel for testing. Household demographic variables included mother's age, mother's highest level of education, household size, floor construction, electricity access, livestock ownership, drinking water source, wealth quintile, indoor residual spraying (IRS) received in the last 12 months, and whether there is a community health worker (CHW) who distributes malaria medicines in the community. Rurality (refugee, urban, or rural cluster) was determined by the survey design. Environmental and geospatial covariates included altitude (meters [m]), average rainfall (millimeters [mm]), aridity index, enhanced vegetation index, proximity to water (m), and cattle density (per square kilometer). December average temperature (°C), January average temperature (°C), land surface temperature (°C), diurnal average temperature (°C), and region-level malaria prevalence measured by microscopy were also included. Detailed variable definitions are available in the supplement (Supplementary Table 1).

### Statistical Analysis

The outcome of interest was the prevalence of false-positive mRDT results, defined as a positive mRDT result and negative microscopy result, among microscopy-negative individuals in each stratum of the covariates listed above. The complex 2-stage sample design was accounted for in all analyses. We reported prevalence estimates and corresponding 95% Wald confidence intervals. Spearman's rank correlation coefficient was calculated between false-positive mRDT prevalence among microscopy-negative children and malaria prevalence measured by mRDT and microscopy among all children at the region level. We constructed locally estimated scatterplot smoothing curves for the observed relationships between false-positive mRDT prevalence and 1) malaria prevalence as measured by microscopy and mRDT, 2) child's age in months, and 3) environmental covariates of interest.

We fit bivariate generalized linear models to estimate the prevalence of false-positive mRDTs among microscopy-negative individuals for each level of the child demographic, clinical and household variables. Prevalence differences (PD) were also estimated for each covariate.

Next, we constructed weighted least absolute shrinkage and selection operator (lasso) regression models [13] with nested 5-fold cross validation that accounts for the survey design [14, 15] to determine which set of variables best predicted false-positive mRDTs among microscopy-negative children with recent fever by estimating the cross-validated weighted area under the receiver operating characteristic curve (AUC). First, we fit a naive model with 1 predictor: region-level malaria prevalence measured by microscopy. Then, we chose 3 sets of candidate predictors, described above. The first included child clinical and demographic variables, which could be reasonably ascertained in a care-seeking setting (clinical model). The second included household demographic characteristics (household model). The third included environmental predictors, chosen to represent differences in malaria transmission intensity across Uganda (environmental model). The full model included all candidate predictors from these sets. The model with the largest weighted AUC was chosen as the final model, unless a more parsimonious model could be achieved with 1% tolerance. We evaluated sensitivity/specificity tradeoffs of the full model at 5 sensitivity thresholds: 5%, 25%, 50%, 75%, and 95%.

For the bivariate and lasso models, we used multiple imputation with fully conditional specification to impute missing values. Most missing data arose from the absence of a linked women's questionnaire for a particular child (Supplementary Table 2), specifically, whether a child had a fever in the last 2 weeks (16.4%), and the mother's age and education level (16.3%). On the household questionnaire, missing data included whether the household received IRS in the last 12 months (0.6%) and whether there is a CHW in the community (3.5%). For geospatial covariates, which are measured at the cluster-level, missing data were present for all covariates, ranging from 1.1% to 10.6% (Supplementary Table 4). For the bivariate models, variances were estimated using the Taylor series linearization method and pooled using Rubin's formula or nested Rubin's formula [16]. More details regarding statistical analyses are available in the supplement. All analyses were conducted using SAS Studio 3.8 and R 4.3.1.

### Ethics

This study uses secondary data from the Demographic and Health Surveys (DHS) Program. The original study protocols were approved by the Uganda National Council for Science & Technology, the Makerere University School of Medicine and Ethics Committee, and the institutional review board at ICF International [17]. Respondents gave consent prior to participation. Guardians provided consent for malaria and anemia testing in children. This secondary analysis was determined to be exempt from review by The University of North Carolina at Chapel Hill Institutional Review Board.

## RESULTS

A total of 8125 under-5s were sampled and 8002 were included in the de facto population (Supplementary Figure 1). Among them, 248 (3.1%) did not have paired microscopy and mRDT results. The remaining 7754 children (96.9% of the sampled population) were included in the analyses. About half (49.7%) of the children were female and 62.2% were reported to have slept under a LLIN the night before the survey (Table 1). More children lived in rural areas than urban areas (70.1% vs 20.7%). The remaining 9.1% lived in refugee settlements. Malaria prevalence as measured by microscopy and mRDT was 9.5% and 18.3%, respectively. Over one quarter (27.7%) of children had a fever, as reported by their guardian, in the 2 weeks prior to the survey. Among them, 74.4% sought care and 62.7% took antimalarials for the fever. Fewer (13.6%) took antibiotics and about half (51%) took antipyretics.

**Table 1.** Demographic and Clinical Characteristics of 7754 Children Under 5 Years of Age With Valid Microscopy and Malaria Rapid Diagnostic Test (mRDT) Results. Unweighted n and Weighted %, Weighted Mean (SD) are Presented

	N (%)
Child demographic characteristics	
Age in m [mean (SD)]	29.9 (0.3)
Age category	…
0–11 m	1559 (19.9)
1 y	1482 (18.8)
2 y	1520 (19.3)
3 y	1603 (21.2)
4 y	1590 (20.8)
Female sex	3820 (49.7)
Slept under LLIN last night	4855 (62.2)
Child clinical characteristics	
Malaria rapid diagnostic test+	1645 (18.3)
Malaria microscopy+	878 (9.5)
*P. falciparum*^a^	782 (89.4)
*P. ovale*^a^	6 (0.4)
*P. malariae*^a^	28 (2.4)
Mixed infection^a^	62 (7.7)
Fever in last 2 wk	1899 (27.7)
Missing	1328
Among children with fever in last 2 wk:	…
Care seeking for fever	1422 (74.4)
Blood taken from finger or heel	1014 (51.0)
Missing	2
Antimalarials taken	1226 (62.7)
Antibiotics taken	259 (13.6)
Antipyretics taken	922 (51.0)
Anemia (Hb < 11 g/dL)	3985 (51.3)
Moderate anemia (Hb < 8 g/dL)	308 (3.6)
Maternal demographic characteristics	
Age	…
15–24 y	2007 (30.1)
25–34 y	3090 (48.6)
35 + y	1335 (21.3)
Missing	1322
Education	…
None	1371 (18.7)
Primary	3684 (56.0)
Secondary	1146 (21.2)
Tertiary/University	231 (4.2)
Missing	1322
Household demographic characteristics	
Number of household members (mean [SD])	6.7 (0.1)
Number of household members	…
2–4	1863 (23.7)
5–6	2352 (31.1)
7–8	1790 (22.5)
9+	1749 (22.7)
Rurality	…
Urban	1478 (20.7)
Rural	5604 (70.1)
Refugee	672 (9.1)
Main floor material	…
Natural	5846 (68.6)
Finished	1908 (31.4)
Has electricity	2758 (39.0)
Own livestock, herds, or farm animals	4949 (61.9)
Source of drinking water	…
Piped	1374 (18.3)
Dug well	1276 (17.5)
Tube well	3750 (46.5)
Surface	1222 (16.1)
Other	132 (1.7)
Household wealth index	…
Poorest	2670 (27.2)
Poorer	1776 (22.5)
Middle	1255 (18.5)
Richer	1072 (16.8)
Richest	981 (15.0)
Indoor residual spraying (IRS) in last 12 m	1015 (11.1)
Missing	41
Community health worker (CHW) distributes antimalarials in community	4772 (56.6)
Missing	259

^a^Among microscopy-positive individuals.

**Figure 1.**
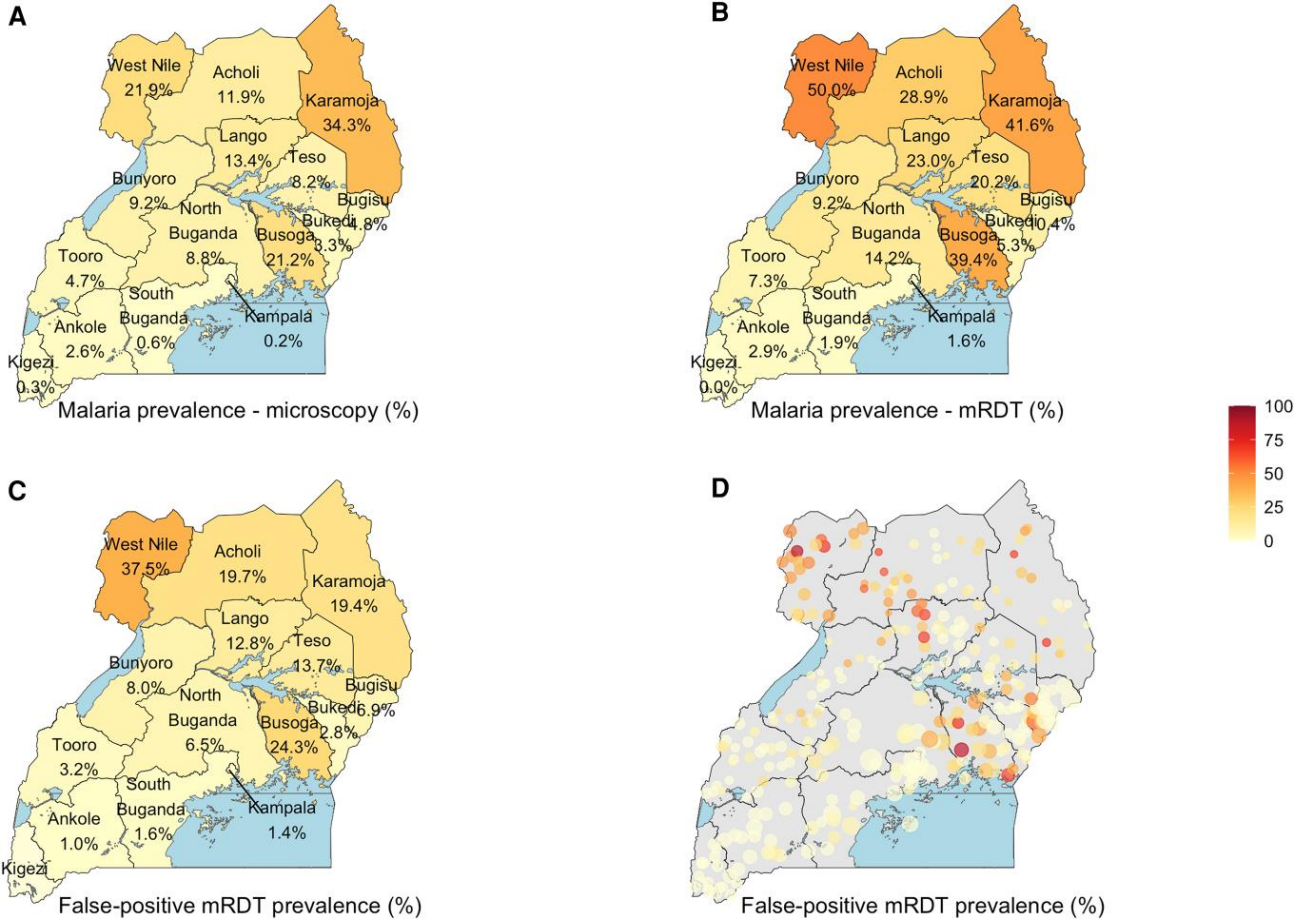
Maps of the 15 regions of Uganda showing malaria prevalence by microscopy *A*, malaria prevalence by mRDT *B*, and false-positive mRDT prevalence among microscopy-negative children *C*. Map of MIS clusters showing false-positive mRDT prevalence among microscopy-negative children *D*. Cluster locations are randomly displaced. The point size reflects the cluster weight. Refugee settlements are excluded. Latitude and longitude are missing for 2 clusters (not shown).

**Figure 2.**
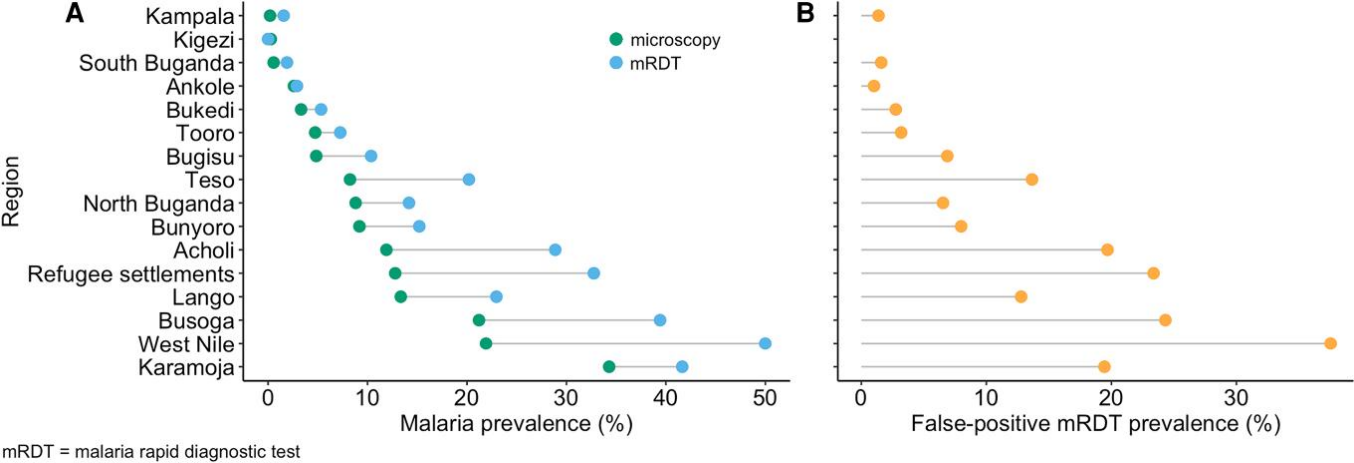
Observed malaria prevalence as measured by microscopy and mRDT *A*, and observed false-positive malaria rapid diagnostic test (mRDT) prevalence among microscopy-negative children *B* for all 15 regions and refugee settlements.

False-positive malaria prevalence tended to increase with increasing malaria prevalence (by mRDT or microscopy), though this trend was nonlinear, and varied by region (Figure 1, Figure 2). The overall prevalence of false-positive mRDT results among the 6876 microscopy-negative under-5s was 10.7% (95% CI: 8.8%, 12.6%), but varied greatly across regions, ranging from 1.0% (0.0%, 2.1%) in Ankole to 37.5% (28.3%, 46.8%) in West Nile (Supplementary Table 3, Figure 1). False-positive mRDT prevalence was higher in rural areas (11.4%) than urban areas (3.4%). Correlation between mRDT and microscopy positivity was 0.98. There was a strong correlation between false-positive mRDT prevalence and malaria prevalence as measured by both mRDT (*r* = 0.96) and microscopy (*r* = 0.9) (Supplementary Figure 2). Within regions, variation existed in false-positive mRDT prevalence by cluster (Figure 1). Slightly less than half of clusters (155/340, 45.6%) did not have any positive mRDT results. In general, cluster-level false-positive mRDT prevalence increased with increasing temperature and proximity to water, and decreased with increasing altitude, aridity index, and cattle density (Supplementary Figure 3).

**Figure 3.**
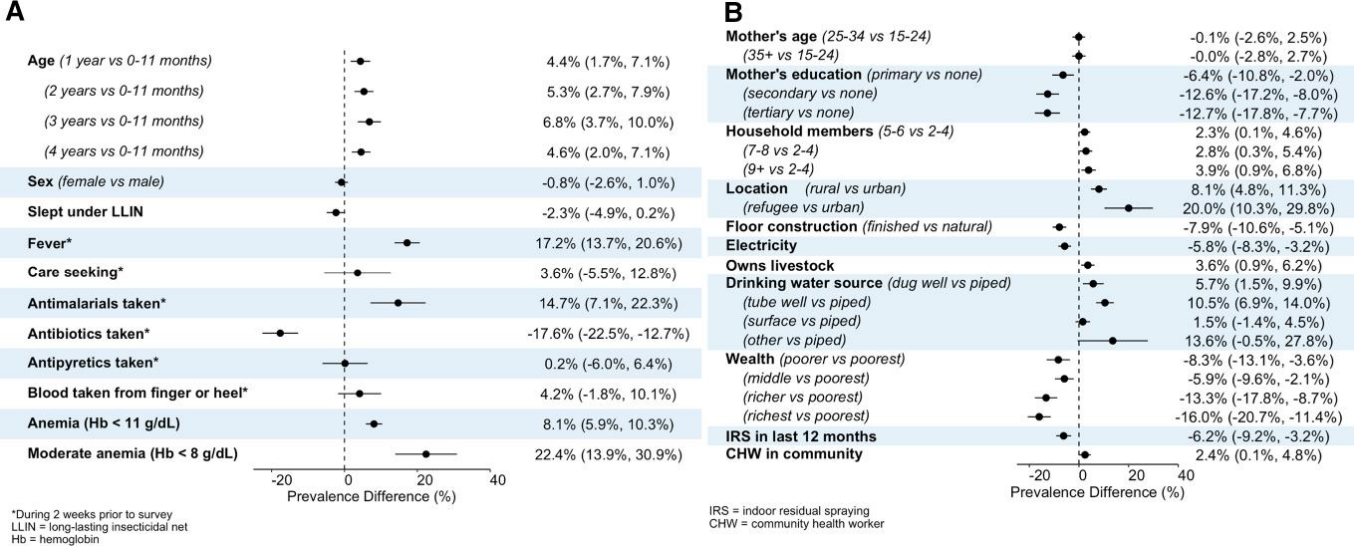
Forest plot of prevalence differences of false-positive mRDTs among microscopy-negative children with corresponding 95% CIs for child demographic and clinical characteristics *A* and mother and household characteristics *B*. Comparisons are listed in italics; otherwise, comparisons are yes versus no.

The prevalence of false-positive mRDT results among microscopy-negative under-5s varied by certain subgroups. This prevalence was lowest among children aged 0–11 months. Prevalence was highest among children aged 3 years, but similar among all children over 1 year (Figure 3, Supplementary Figure 4). Children with reported fever in the 2 weeks prior to the survey had a higher prevalence of false-positive mRDTs compared to those without (PD: 17.2%, 95% CI: (13.7%, 20.6%)) (Figure 3, Supplementary Figure 5). Among children with recent fever, the prevalence of false-positive mRDTs was approximately 15 percentage points higher among those who took antimalarials than those who did not, and nearly 18 percentage points lower among those who took antibiotics compared to those who did not. Children who sought care in the 2 weeks prior to the survey had a slightly higher false-positive mRDT prevalence than those who did not, while those who had blood drawn for testing care in the 2 weeks prior to the survey had a slightly lower false-positive mRDT prevalence than those who did not. Certain household demographic factors were associated with increased false-positive mRDT prevalence, including residing in a rural cluster or refugee settlement, lower maternal education, and lower household wealth (Figure 3, Supplementary Figure 5).

**Figure 4.**
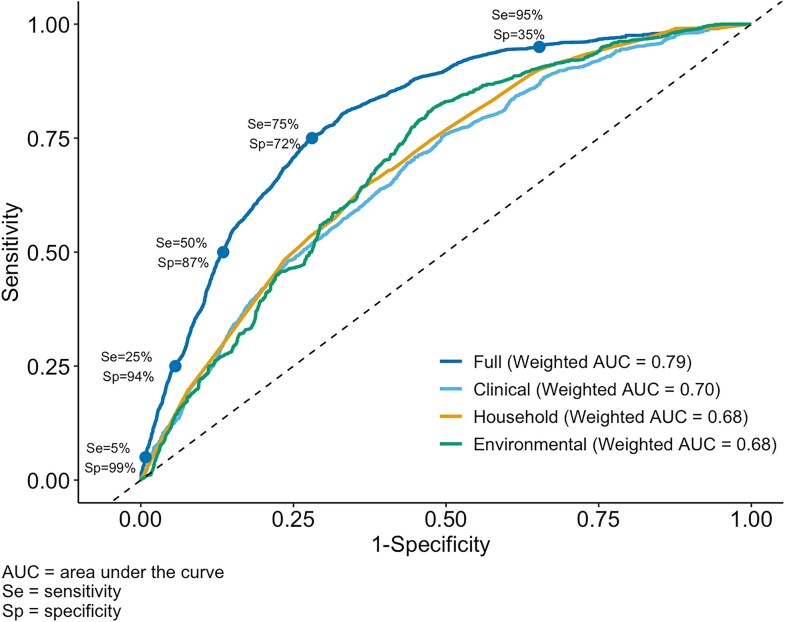
Receiver operating characteristic (ROC) curves assessing model performance and corresponding cross-validated weighted area under the receiver operating characteristic curve (AUC) from lasso models with different sets of candidate predictors. Sensitivity (Se) and specificity (Sp) thresholds are shown for the full model.

**Figure 5.**
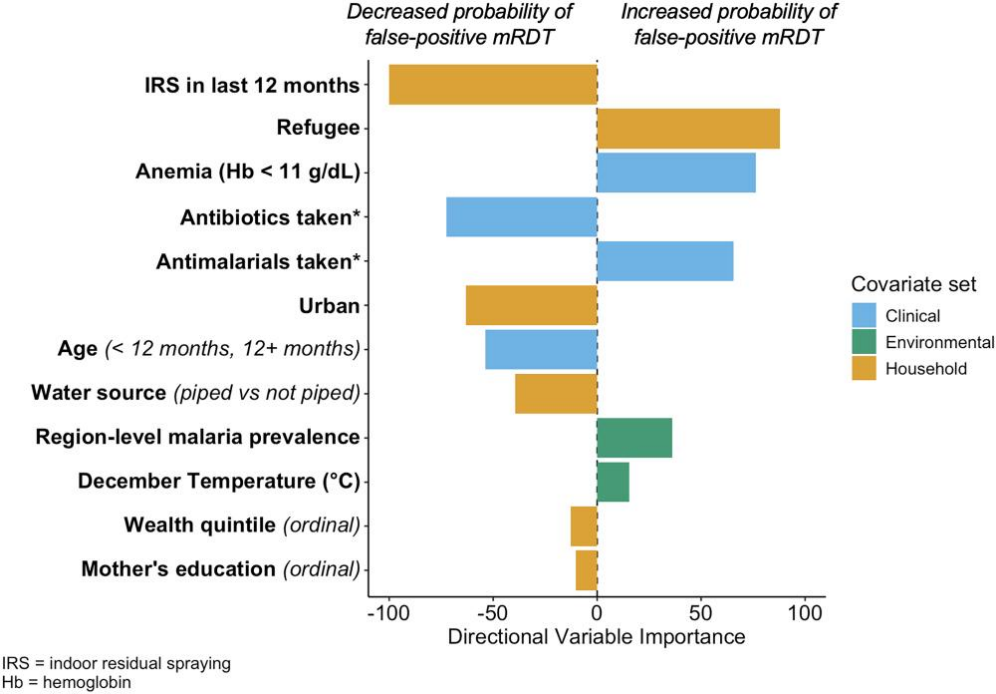
Directional variable importance of retained variables from lasso model with full set of candidate covariates. The variable importance is the absolute magnitude. The most important variable has importance of 100 or −100. All other variables are ranked relative to the most important. Variable importance less than 0 indicates that the covariate is associated with a decreased probability of a false-positive mRDT. Variable importance >0 indicates that the covariate is associated with an increased probability of a false-positive mRDT.

The full lasso model containing clinical, environmental, and household covariates restricted to children who had a fever in the 2 weeks prior to the survey performed best among candidate models in distinguishing those with and without a false-positive mRDT with a cross-validated weighted AUC of 0.79 (Figure 4). In other lasso models restricted to children with recent fever, the naive model (weighted AUC: 0.69), model with environmental covariates (0.68), model with clinical covariates (0.70), and model with household covariates (0.68) performed similarly. In the environmental model, 2 variables were retained (altitude and region-level malaria prevalence) (Supplementary Figure 6). In the clinical model, 4 variables were retained, with anemia (Hb < 11 g/dL) having the highest variable importance. In the household model, 6 variables were retained with IRS in the last 12 months having the highest variable importance. The full model retained 12 variables, including those from all 3 sets of candidate predictors (Figure 5). All variables retained in the clinical and household models were retained in the full model. However, altitude was not retained, instead December temperature and region-level malaria prevalence were retained. In the full model, the covariates with highest variable importance were 1) IRS in the last 12 months, which decreased the probability of a false-positive mRDT and 2) living in a refugee cluster, which increased the probability of a false-positive mRDT. Model hyperparameter and parameter estimates are shown in Supplementary Table 5.

## DISCUSSION

In the general population of microscopy-negative children under 5 years of age in Uganda, the prevalence of false-positive mRDTs was nearly 11%, indicating that false-positive mRDTs are frequent in this population. The prevalence of false-positive mRDTs was associated with demographic, clinical, and environmental features. Recent fever and antimalarial use were associated with an increased probability of a false-positive mRDT, suggesting that many mRDT-positive results shortly after antimalarial use may be due to antigen persistence. Conversely, recent antibiotic use was associated with a decreased probability of a false-positive mRDT, possibly because antibiotics were used to treat a nonmalarial illness. The prevalence of false-positive mRDTs generally increased with malaria prevalence, suggesting that the consequences of false-positive mRDTs are more pronounced in higher transmission settings. The overestimation of malaria prevalence by mRDT due to false positives in the entire UMIS sample may have important consequences for the allocation and prioritization of resources for malaria control, especially in higher transmission settings, where mRDT prevalence may greatly overestimate true malaria prevalence.

Among under-5s who had a fever in the 2 weeks prior to the survey, lasso regression models with demographic, clinical, and environmental variables modestly predicted false-positive mRDT results. We used under-5s with recent fever to represent a care-seeking population. Demographic and clinical characteristics could be ascertained by clinicians who treat care-seeking febrile children. Environmental covariates, however, were included to represent malaria transmission intensity. The naive model, containing only region-level malaria prevalence to represent transmission intensity, performed worse than the full model. Further, in the full model, 8 covariates had greater variable importance than region-level malaria prevalence, suggesting that factors beyond local transmission intensity should be considered when determining whether a patient may be more likely to have a false-positive mRDT. In this scenario, the overestimation of malaria prevalence may lead to misdiagnosis, under-recognition of nonmalarial febrile illness, and antimalarial overprescription, even when treatment algorithms are correctly followed.

Our results generally align with the limited existing literature. False-positive mRDTs have been noted in a range of care-seeking settings in Uganda [18–20]. Previous work showed that children tend to remain mRDT-positive longer than adults [8]. Here, we found that the youngest children (under one year of age) are less likely to have false-positive mRDTs than those aged 1 to <5 years. Additionally, in Uganda, prior work demonstrated that the specificity of mRDTs is lower in high transmission areas compared to low transmission areas, suggesting a relationship between false-positive mRDT prevalence and transmission intensity [9]. We found that other variables known to be associated with higher malaria transmission (eg, rural clusters and less wealth) were associated with increased false-positive mRDT prevalence. Higher parasitemia at time of testing has been associated with longer antigen persistence [9, 10]. In this study, we were unable to evaluate the relationship between parasitemia and false-positive mRDT prevalence because parasite density was not reported.

Factors other than antigen persistence may be associated with or cause false-positive mRDTs, including the test brand, cross-reactivity, temperature, humidity, and user error [4, 21]. These sources of measurement error may exist in the UMIS, but we cannot distinguish these errors from antigen persistence. In addition, we assumed that there was perfect measurement and reporting of malaria testing results by mRDT and microscopy in the UMIS, as testing was performed by multiple trained fieldworkers. However, there may have been misclassification of these results as variation can exist between individuals who read the rapid tests and microscopy slides. Further, our definition of false-positive mRDTs (positive mRDT and negative microscopy) may be subject to limitations. While microscopy is the gold standard for identifying malaria infection in Uganda [22], it is less sensitive than PCR and may be subject to false-negative results caused by low parasite densities [23]. However, false-negative results are expected to be infrequent in our study sample because the prevalence of *pfhrp2* gene deletions in *P. falciparum* in Uganda was low at the time of the survey [11, 24, 25].

These results may not be generalizable to older children or adults, or other populations in SSA. Malaria prevalence measured by microscopy exceeded malaria prevalence measured by mRDT in only 7/55 cross-sectional MIS and DHS surveys estimating malaria prevalence in children under 5 from 2006 to 2024. These 7 surveys were conducted in 4 countries: Benin, Madagascar, Mali and Senegal [26]. Our findings related to the sizeable prevalence of false-positive mRDTs may not be relevant in populations where microscopy prevalence exceeds mRDT prevalence. Crucially, these findings may also not reflect the false-positive mRDT prevalence in care-seeking populations, as the UMIS was designed to survey children in the general population. The results may also not be generalizable to regions where *P. falciparum* is not the primary malaria species, as HRP-2 is specific to *P. falciparum,* or where *P. falciparum* is the primary species, but mRDTs that target other antigens are used. Further, general limitations of self-reported questionnaire data may apply. Responses provided by women and household members during interviews may be subject to recall bias. Most environmental and geospatial covariates were collected prior to the UMIS and may not reflect conditions during the survey. Further, there may exist important correlates with false-positive mRDT probability that were unmeasured and not evaluated in this study. Lastly, the survey's cross-sectional design means that we were unable to assess any causal associations between covariates and false-positive mRDTs.

Much of prior work on malaria infection misclassification involves false-negative mRDTs [27]. Here, we demonstrate that there is a sizeable burden of false-positive mRDTs among under-5s in Uganda. These false-positive mRDTs may pose consequences for malaria case management and elimination efforts by misguiding the allocation of resources. While the introduction of mRDTs into health facilities and care-seeking settings decreased antimalarial use [28–32] and reduced antimalarial overprescription [18, 33–37], false-positive mRDTs may still contribute to antimalarial overprescription among under-5s in high transmission areas of Uganda, despite adherence to testing and treatment algorithms by healthcare workers. In Uganda, where malaria is highly burdensome, even small proportions of antimalarial overprescription due to false-positive mRDTs may impact emerging drug resistance and misdiagnosis of nonmalarial febrile illnesses. Two-step diagnostic algorithms that use microscopy as a confirmatory test for certain mRDT results have been proposed [38], which could reduce the amount of microscopy performed. Based on our results, certain clinical features, such as anemia (Hb < 11 g/dL) and recent antimalarial use, as well as environmental features, such as residing in a high transmission setting, might suggest that mRDT-positive children who seek care are more likely to be false positives. Further work is needed to determine whether existing treatment algorithms could be modified to use covariates that predict mRDT false-positivity by utilizing a child's clinical history, in conjunction with a positive mRDT, to determine whether antimalarials should be provided.

## Supplementary Material

jiaf604_Supplementary_Data

## Supplementary Data

Supplementary materials are available at *The Journal of Infectious Diseases* online (http://jid.oxfordjournals.org/). Supplementary materials consist of data provided by the author that are published to benefit the reader. The posted materials are not copyedited. The contents of all supplementary data are the sole responsibility of the authors. Questions or messages regarding errors should be addressed to the author.

## Notes

***Data Availability***. Data available at https://dhsprogram.com/.

***Financial support.*** This work was supported by awards from the National Institutes of Health (T32AI070114 to C. A. C., K01AI182506 to B. E. S, K23AI173658 to E. J. C, and R21MH125705 to A. M. Y.).

## References

[1] Aidoo M, Incardona S. Ten years of universal testing: how the rapid diagnostic test became a game changer for malaria case management and improved disease reporting. Am J Trop Med Hyg 2022; 106:29–32. 34749303 10.4269/ajtmh.21-0643 PMC8733544

[2] Boyce MR, O’Meara WP. Use of malaria RDTs in various health contexts across sub-Saharan Africa: a systematic review. BMC Public Health 2017; 17:470. 28521798 10.1186/s12889-017-4398-1 PMC5437623

[3] Centers for Disease Control and Prevention. Blood Specimens – Detection of Parasite Antigens. 2016 [cited 2024 Aug 29] Available at: https://www.cdc.gov/dpdx/diagnosticprocedures/blood/antigendetection.html.

[4] Watson OJ, Sumner KM, Janko M, et al False-negative malaria rapid diagnostic test results and their impact on community-based malaria surveys in sub-Saharan Africa. BMJ Global Health 2019; 4:e001582. 10.1136/bmjgh-2019-001582 PMC6666813 31406591

[5] Lamsfus Calle C, Schaumburg F, Rieck T, et al Slow clearance of histidine-rich protein-2 in Gabonese with uncomplicated malaria. Microbiol Spectrum 2024; 0:e00994-24. 10.1128/spectrum.00994-24 PMC11449231 39194289

[6] Dondorp AM, Desakorn V, Pongtavornpinyo W, et al Estimation of the total parasite biomass in acute falciparum malaria from plasma PfHRP2. PLoS Med 2005; 2:e204. 16104831 10.1371/journal.pmed.0020204 PMC1188247

[7] Marquart L, Webb L, O’Rourke P, et al The in-vivo dynamics of Plasmodium falciparum HRP2: implications for the use of rapid diagnostic tests in malaria elimination. Malar J 2022; 21:233. 35922803 10.1186/s12936-022-04245-z PMC9351188

[8] Dalrymple U, Arambepola R, Gething PW, Cameron E. How long do rapid diagnostic tests remain positive after anti-malarial treatment? Malar J 2018; 17:228. 29884184 10.1186/s12936-018-2371-9 PMC5994115

[9] Grandesso F, Nabasumba C, Nyehangane D, et al Performance and time to become negative after treatment of three malaria rapid diagnostic tests in low and high malaria transmission settings. Malar J 2016; 15:1–12. 27716244 10.1186/s12936-016-1529-6 PMC5050565

[10] Tjitra E, Suprianto S, McBroom J, Currie BJ, Anstey NM. Persistent ICT malaria P.f/P.v panmalarial and HRP2 antigen reactivity after treatment of plasmodium falciparum malaria is associated with gametocytemia and results in false-positive diagnoses of plasmodium vivax in convalescence. J Clin Microbiol 2001; 39:1025–31. 11230422 10.1128/JCM.39.3.1025-1031.2001 PMC87868

[11] World malaria report 2023 . Geneva: World Health Organization; 2023.

[12] World malaria report 2022 . Geneva: World Health Organization; 2022.

[13] Iparragirre A, Lumley T, Barrio I, Arostegui I. Variable selection with LASSO regression for complex survey data. Stat 2023; 12:e578.

[14] Wieczorek J, Guerin C, McMahon T. K-fold cross-validation for complex sample surveys. Stat 2022; 11:e454.

[15] Cawley GC, Talbot NL. On over-fitting in model selection and subsequent selection bias in performance evaluation. J Mach Learn Res 2010; 11:2079–107.

[16] Harel O, Schafer JL. Multiple imputation in two stages. In: Proceedings of federal committee on statistical methodology 2003 conference; 2003; 126.

[17] Uganda National Malaria Control Division (NMCD), Uganda Bureau of Statistics (UBOS), and ICF. Uganda Malaria Indicator Survey 2018–19. Kampala, Uganda, and Rockville, Maryland, USA: NMCD, UBOS, and ICF; 2020.

[18] Kyabayinze DJ, Asiimwe C, Nakanjako D, Nabakooza J, Counihan H, Tibenderana JK. Use of RDTs to improve malaria diagnosis and fever case management at primary health care facilities in Uganda. Malar J 2010; 9:1–10. 20624312 10.1186/1475-2875-9-200 PMC2914063

[19] Mortazavi SE, Lugaajju A, Ivarsson AC, Karlsson Söbirk S, Norrgren H, Persson KEM. High rate of false positive malaria rapid diagnostic tests in a district hospital in Uganda. Front Malar 2025; 3:1545825.

[20] Mbabazi P, Hopkins H, Osilo E, Kalungu M, Byakika-Kibwika P, Kamya MR. Accuracy of two malaria rapid diagnostic tests (RDTS) for initial diagnosis and treatment monitoring in a high transmission setting in Uganda. Am J Trop Med Hyg 2015; 92:530–6. 25624399 10.4269/ajtmh.14-0180 PMC4350543

[21] Maltha J, Gillet P, Jacobs J. Malaria rapid diagnostic tests in endemic settings. Clin Microbiol Infect 2013; 19:399–407. 23438048 10.1111/1469-0691.12151

[22] The Republic of Uganda Ministry of Health . Uganda National Malaria Control Policy. 2011 Jun [cited 2023 Sep 10]. Available at: https://library.health.go.ug/sites/default/files/resources/Uganda%20National%20Malaria%20Control%20Policy%202011.pdf.

[23] Roth JM, Korevaar DA, Leeflang MMG, Mens PF. Molecular malaria diagnostics: a systematic review and meta-analysis. Critic Rev Clin Lab Sci 2016; 53:87–105. 10.3109/10408363.2015.1084991 26376713

[24] Agaba BB, Smith D, Travis J, et al Limited threat of Plasmodium falciparum pfhrp2 and pfhrp3 gene deletion to the utility of HRP2-based malaria RDTs in Northern Uganda. Malar J 2024; 23:3. 38167003 10.1186/s12936-023-04830-w PMC10759665

[25] Nsobya SL, Walakira A, Namirembe E, et al Deletions of pfhrp2 and pfhrp3 genes were uncommon in rapid diagnostic test-negative plasmodium falciparum isolates from Uganda. Malar J 2021; 20:4. 33386076 10.1186/s12936-020-03547-4 PMC7777526

[26] ICF . The DHS Program STATcompiler. Funded by USAID [Internet]. 2015 [cited 2025 Oct 17]. Available at: http://www.statcompiler.com.

[27] Thomson R, Beshir KB, Cunningham J, et al Pfhrp2 and pfhrp3 gene deletions that affect malaria rapid diagnostic tests for plasmodium falciparum: analysis of archived blood samples from 3 African countries. J Infect Dis 2019; 220:1444–52. 31249999 10.1093/infdis/jiz335 PMC6761929

[28] Bruxvoort K, Kalolella A, Nchimbi H, et al Getting antimalarials on target: impact of national roll-out of malaria rapid diagnostic tests on health facility treatment in three regions of Tanzania. Trop Med Int Health 2013; 18:1269–82. 23937722 10.1111/tmi.12168 PMC4282336

[29] Masanja IM, Selemani M, Amuri B, et al Increased use of malaria rapid diagnostic tests improves targeting of anti-malarial treatment in rural Tanzania: implications for nationwide rollout of malaria rapid diagnostic tests. Malar J 2012; 11:221. 22747655 10.1186/1475-2875-11-221 PMC3471012

[30] Thiam S, Thior M, Faye B, et al Major reduction in anti-malarial drug consumption in Senegal after nation-wide Introduction of malaria rapid diagnostic tests. PLoS One 2011; 6:e18419. 21494674 10.1371/journal.pone.0018419 PMC3071817

[31] Burchett HED, Leurent B, Baiden F, et al Improving prescribing practices with rapid diagnostic tests (RDTs): synthesis of 10 studies to explore reasons for variation in malaria RDT uptake and adherence. BMJ Open 2017; 7:e012973. 10.1136/bmjopen-2016-012973 PMC5353269 28274962

[32] Yukich JO, Bennett A, Albertini A, et al Reductions in artemisinin-based combination therapy consumption after the nationwide scale up of routine malaria rapid diagnostic testing in Zambia. Am J Trop Med Hyg 2012; 87:437–46. 22848096 10.4269/ajtmh.2012.12-0127 PMC3435345

[33] Williams PCM, Isaacs D, Berkley JA. Antimicrobial resistance among children in sub-Saharan Africa. Lancet Infect Dis 2018; 18:e33-44. 29033034 10.1016/S1473-3099(17)30467-X PMC5805911

[34] Ansah EK, Narh-Bana S, Affran-Bonful H, et al The impact of providing rapid diagnostic malaria tests on fever management in the private retail sector in Ghana: a cluster randomized trial. BMJ 2015; 350:h1019. 25739769 10.1136/bmj.h1019 PMC4353311

[35] Shelus V, Mumbere N, Mulogo EM, et al Private sector antimalarial sales a decade after “test and treat”: a cross-sectional study of drug shop clients in rural Uganda. Front Public Health 2023; 11:1140405. 37056663 10.3389/fpubh.2023.1140405 PMC10089286

[36] Bruxvoort KJ, Leurent B, Chandler CIR, et al The impact of introducing Malaria rapid diagnostic tests on fever case management: a synthesis of ten studies from the ACT Consortium. 2017 Aug 7 [cited 2024 Jul 3]; Available at: https://www.ajtmh.org/view/journals/tpmd/97/4/article-p1170.xml. 10.4269/ajtmh.16-0955 PMC5637593 28820705

[37] Ansah EK, Narh-Bana S, Epokor M, et al Rapid testing for malaria in settings where microscopy is available and peripheral clinics where only presumptive treatment is available: a randomised controlled trial in Ghana. BMJ 2010; 340:c930. 20207689 10.1136/bmj.c930 PMC2833239

[38] Murungi M, Fulton T, Reyes R, et al Improving the specificity of plasmodium falciparum malaria diagnosis in high-transmission settings with a two-step rapid diagnostic test and microscopy algorithm. J Clin Microbiol 2017; 55:1540–9. 28275077 10.1128/JCM.00130-17 PMC5405272

