# Supplementary material for: False-positive Malaria Rapid Diagnostic Tests are Prevalent Among Children Under 5 Years of Age in Uganda
Source: J Infect Dis. 2025 Nov 28;233(3):e782–90. doi: 10.1093/infdis/jiaf604 (PMC12880570; doi:10.1093/infdis/jiaf604)
Supplement: jiaf604_Supplementary_Data [file jiaf604_supplementary_data.zip › supplement_17NOV25_clean.docx]

**Supplemental methods**

Further details about the MIS

*Design:*

The most recent Uganda MIS took place from December 2018 to January 2019. The Uganda MIS is a nationally representative complex survey designed to estimate national and regional malaria prevalence among children under 5, among other factors.[1] The data are publicly available from the DHS program.[2] Households were sampled for inclusion, and a household member was interviewed. All women aged 15-49 in sampled households were eligible for an additional interview, referred to as the "women's questionnaire". All children aged 0-59 months in sampled households were eligible for biomarker testing, including microscopy and mRDT for malaria, and hemoglobin (Hb) for anemia, defined as Hb <11 g/dL. Interviews were conducted by teams of fieldworkers, where at least one fieldworker was a nurse who performed biomarker testing and provided medication to children according to treatment protocols. Selected households were included in the sample if at least one adult household member was present at the time of interview. Notably, the response rates were high: 99% of eligible households occupied at the time of fieldwork were interviewed, 98% of eligible women were interviewed, and 97% of eligible children were tested for malaria and anemia.

*Sampling procedures:*

Participants were sampled in a two-stage sampling procedure from all 15 regions of Uganda. The sampling frame used in the first stage was a complete list of enumeration areas derived from the 2014 Uganda Population and Housing Census and in the second stage was the list of all households in each cluster which was derived during field visits to the selected cluster. In the first stage, 16 strata were created, representing 15 regions and refugee settlements. Then, 84 urban and 236 rural clusters, as well as 22 refugee settlements, were selected using a probability proportional to size (PPS) selection procedure from these strata. Each stratum contained at least one rural and one urban cluster, except Kampala, which contained only urban clusters. The number of clusters allocated per stratum ranged from 18 to 27. In the second stage, 28 households were selected per cluster using systematic sampling without replacement. Urban areas within strata and strata with small populations were oversampled. Clusters represent the primary sampling units (PSUs) and households represent the secondary sampling units (SSUs).

*Sampling weights:*

MIS sampling weights were derived as the inverse probability of selection in the two stages, by multiplying a weight for the probability of selecting a cluster in a particular stratum and a weight for the probability of selecting a household in a particular cluster. Weights were then adjusted for household and individual non-response and calibrated to national population totals from the 2014 National Population and Housing Census. Individual weights are provided by the DHS for household questionnaire and the women's questionnaire. For cluster-level analyses, cluster weights were approximate using guidance from the DHS.[3]

*Malaria testing:*

mRDT testing was performed by trained field workers using the SD BIOLINE Malaria Ag *P.f.* (HRP-II) mRDT for all participants. Blood smears were collected in the field during the survey and transported to a laboratory for reading after the survey period. Standard DHS protocols were used to read blood slides for microscopy. Slides were stained with Giemsa and read by at least two trained laboratory technicians. A negative result was determined if no parasites were detected after counting 200 fields. A positive result was determined if any parasites were detected.

*Environmental covariates:*

Environmental and cluster-level variables were estimated by the DHS Program Geospatial Team using data from various sources.[4] All variables were estimated within the 2 km (urban) or 10 km (rural) buffer surrounding the DHS survey cluster.

*Maps:*

Coordinates for MIS clusters were provided by DHS[2] and are randomly displaced to ensure confidentiality. Urban clusters are displaced up to 2 km, rural clusters and displaced up to 5 km, with 1% of rural clusters displaced up to 10 km. Shapefiles were obtained from the Uganda Bureau of Statistics[5] and DHS[6].

Prevalence estimates

The complex two-stage sample design was accounted for in all analyses. We reported prevalence estimates and corresponding 95% Wald confidence intervals with variance estimated using the Taylor series linearization method and domain estimation.

Multiple imputation

Missing covariate data was imputed using multiple imputation by chained equations using the *mice* R package[7] prior to conducting bivariate and lasso regression analyses. All variables included in table S1 were included in the imputation model. The Classification and Regression Trees, 'cart', method was used to overcome multicollinearity in the data. 30 imputations with 30 iterations were conducted. Trace plots were assessed to confirm model convergence.

A two-stage extension of multiple imputation was used to impute missing values for the variables denoting whether a child received antimalarials, antibiotics, antipyretics, sought care for fever, or had blood taken for testing in the 2 weeks prior to the survey. These questions were only asked in the MIS for children whose guardian reported a fever in the 2 weeks prior to the survey. Thus, we needed to impute these variables only among children with fever. In the first stage, values for fever were imputed for all observations resulting in 30 imputations. Then, in the second stage, for each of the previously imputed datasets, we restricted to observations with a fever. Then, we imputed values for the five variables, using logistic regression, resulting in 30 imputations. 900 imputations were used for variables with two-stage imputation. The number of missing observations for survey data and geospatial data are provided in Tables S3 and S4.

Bivariate analyses

The complex survey design, including strata, cluster, and weights, was accounted for using the *survey* R package.[8] For each imputed dataset, a linear binomial model with a linear link function and a quasibinomial distribution to accommodate survey weights was fit. For single-stage imputation variables, the results of the imputations were pooled, and the variance was estimated using Taylor Series Linearization and pooled using Rubin's formula. For two-stage imputation variables, the results of the imputations were pooled using the nested Rubin's formula.[9] Variances were estimated using Taylor Series Linearization.

Lasso regression models

Because this method is more computationally intense, fewer imputed datasets were used in the weighted lasso regression analysis. 10 imputations were conducted in each stage, for a total of 100 imputations. The imputation framework above was followed, however, among a subset of observations, children with fever. The 100 imputed datasets were concatenated, or "stacked", following the recommendations of Du et al.[10] The sampling weights were divided by the number of imputed datasets. All continuous variables were standardized to have a mean of 0 and a standard deviation of 1.

To handle the 2-stage complex survey design, cross-validation folds were created by stratifying the study sample by the sampling strata and randomly assigning entire clusters to a particular fold.[11] Nested cross-validation was used to avoid overfitting the hyperparameter $\lambda$[12], using the *nestedcv* and *caret* R packages.[13,14] 5 outer folds and 5 inner folds were used.

Model performance was evaluated using a cross-validated weighted AUC (area under the receiver operating characteristic curve)[15]. Weighted receiver operating characteristic (ROC curves) were plotted for the "stacked" dataset using the *PRROC* R package.[16] The model with the largest weighted AUC was selected as the final model, unless a more parsimonious model could be achieved with an AUC within 1% of the largest AUC. If so, that model was selected as the final model. The ROC curves represent sensitivity/specificity pairs of the model at all possible decision thresholds. We evaluated sensitivity/specificity trade-offs of the model at five sensitivity thresholds: 5%, 25%, 50%, 75%, and 95%.

**Supplemental tables**

**Table S1. Variable definitions.** Categorizations are denoted in italics.

| **Variable** | **Definition** |
| --- | --- |
| Rurality | Location of cluster used for sampling (urban, rural, or refugee) |
| *Household questionnaire* | |
| Child's age | Child's age in months (0-59), converted to years |
| Child's sex | Child's sex (male or female) |
| Slept under LLIN | Whether the child slept under a treated or untreated net during the night prior to the survey |
| Anemia | Hemoglobin (Hb) was tested during the household visit portion of the survey by collecting blood from a finger or heel stick and testing with the portable HemoCue device. Levels are adjusted for altitude.  *Anemia defined as adjusted Hb < 11 g/dL*  *Moderate anemia defined as adjusted Hb < 8 g/dL* |
| Household members | Number of de facto household members (individuals who stayed in the household the night prior the survey) |
| Floor construction | Main floor material of dwelling  *Finished = Parquet or polished wood, concrete, ceramic tiles, cement, carpet, stones, or bricks*  *Natural = Earth/sand, dung, wood planks, or palm/bamboo* |
| Electricity | Whether household has electricity |
| Owns livestock | Whether household owns livestock, herds, or farm animals |
| Drinking water source | Source of household drinking water  *Piped = piped into dwelling, piped to yard/plot, public tap/standpipe, or piped to neighbor*  *Tube well = tube well or borehole*  *Dug well = protected well, unprotected well*  *Surface = protected spring, unprotected spring, surface water*  *Other = Tanker truck, rainwater, cart with small tank, other* |
| Household wealth index | Wealth quintiles for entire survey population constructed using household asset data via principal component analysis |
| Indoor residual spraying (IRS) in last 12 months | Whether dwelling has been sprayed against mosquitoes in the last 12 months |
| Community health worker (CHW) in community | Whether there is a community health worker (CHW), community medicine distributor (CMD), or village health team (VHT) who distributes malaria medicines in the community |
| *Women's questionnaire* | |
| Fever | Whether the child had a fever in the 2 weeks preceding the survey |
| Care seeking for fever | Among children with fever in the 2 weeks preceding the survey, whether advice or treatment was sought |
| Antimalarials taken | Among children with fever in the 2 weeks preceding the survey, whether the child took antimalarial drugs |
| Antibiotics taken | Among children with fever in the 2 weeks preceding the survey, whether the child took antibiotic drugs |
| Antipyretics taken | Among children with fever in the 2 weeks preceding the survey, whether the child took aspirin, acetaminophen, or ibuprofen for fever |
| Blood taken from finger or heel | Among children with fever in the 2 weeks preceding the survey, whether children had blood taken from the finger or heel for testing |
| Mother's age | Mother's age in years |
| Mother's education | Highest level of schooling attended |
| *Geospatial Covariates* | |
| Altitude | The cluster's elevation/altitude (in meters) from the SRTM (Shuttle Radar Topography Mission) DEM (Digital Elevation Model) for the specified coordinate location |
| Aridity index^1^ | The ratio of annual precipitation to annual potential evapotranspiration in 2015 |
| Precipitation^1^ | Average precipitation (mm) in 2015 |
| Rainfall^1^ | Average rainfall (mm) in 2015 |
| December temperature^1^ | Average temperature (**°**C) in December, from 1970-2000 |
| January temperature^1^ | Average temperature (**°**C) in January, from 1970-2000 |
| Land temperature^1^ | Average daytime land surface temperature (**°**C) in 2015 |
| Proximity to water^1^ | The geodesic distance to either a lake or the coastline in 2017 in kilometers |
| Enhanced vegetation index^1^ | Average vegetation index value between 0 (least vegetation) and 10000 (Most vegetation) |
| Diurnal temperature range^1^ | Average annual daytime temperature range (**°**C) in 2015 |
| Cattle^1^ | Average density of cattle in 2006 |

^1^within the 2 km (urban) or 10 km (rural) buffer surrounding the DHS survey cluster location

**Table S2. Sample sizes for bivariate analyses among microscopy-negative children.** Missing values were imputed.

|  | **n** |
| --- | --- |
| Overall | 6,876 |
| *Child demographic characteristics* | |
| Age |  |
| 0-11 months | 1,489 |
| 1 year | 1,365 |
| 2 years | 1,313 |
| 3 years | 1,372 |
| 4 years | 1,337 |
| Sex |  |
| Male | 3,498 |
| Female | 3,378 |
| Slept under LLIN last night |  |
| No | 2,453 |
| Yes | 4,423 |
| *Child clinical characteristics* | |
| Fever in last 2 weeks |  |
| No | 4,151 |
| Yes | 1,597 |
| *Missing* | 1,128 |
| Care seeking for fever in last 2 weeks^1^ |  |
| No | 379 |
| Yes | 1,218 |
| Antimalarials taken in last 2 weeks^1^ |  |
| No | 558 |
| Yes | 1,039 |
| Antibiotics taken in last 2 weeks^1^ |  |
| No | 1,365 |
| Yes | 232 |
| Antipyretics taken in last 2 weeks^1^ |  |
| No | 814 |
| Yes | 783 |
| Blood taken from finger or heel in last 2 weeks^1^ |  |
| No | 727 |
| Yes | 869 |
| *Missing* | 1 |
| Anemia (Hb < 11 g/dL) |  |
| No | 3,565 |
| Yes | 3,311 |
| Moderateanemia (Hb < 8 g/dL) |  |
| No | 6,671 |
| Yes | 205 |
| *Maternal demographic characteristics* | |
| Mother's Age |  |
| 15-24 years | 1,832 |
| 25-34 years | 2,754 |
| 35+ years | 1,167 |
| *Missing* | 1,123 |
| Mother's Education |  |
| None | 1,117 |
| Primary | 3,301 |
| Secondary | 1,106 |
| Tertiary/University | 229 |
| *Missing* | 1,123 |
| *Household demographic characteristics* | |
| Number of household members |  |
| 2-4 | 1,710 |
| 5-6 | 2,054 |
| 7-8 | 1,566 |
| 9+ | 1,546 |
| Rurality |  |
| Urban | 1,415 |
| Rural | 4,879 |
| Refugee | 582 |
| Floor construction |  |
| Natural | 5,047 |
| Finished | 1,829 |
| Has electricity |  |
| No | 4,288 |
| Yes | 2,588 |
| Own livestock, herds, or farm animals |  |
| No | 2,535 |
| Yes | 4,341 |
| Source of drinking water |  |
| Piped | 1,313 |
| Dug well | 1,133 |
| Tube well | 3,196 |
| Surface | 1,115 |
| Other | 119 |
| Household wealth index |  |
| Poorest | 2,158 |
| Poorer | 1,587 |
| Middle | 1,141 |
| Richer | 1,019 |
| Richest | 971 |
| Indoor residual spraying (IRS) in last 12 months |  |
| No | 5,868 |
| Yes | 970 |
| *Missing* | 38 |
| Community health worker (CHW) distributes antimalarials in community |  |
| No | 2,502 |
| Yes | 4,130 |
| *Missing* | 244 |

^1^Among children with fever

**Table S3. Malaria prevalence by microscopy, malaria prevalence by mRDT, and false-positive mRDT prevalence among microscopy-negative children overall, by region, and by rurality.** Results are presented as mean (95% CI).

|  | Malaria (microscopy)  n=7,754 | Malaria (mRDT)  n=7,754 | False-positive mRDT  n=6,876 |
| --- | --- | --- | --- |
| Overall |  |  |  |
|  | 9.46 (7.77, 11.15) | 18.32 (15.46, 21.18) | 10.68 (8.80, 12.56) |
| By Region |  |  |  |
| Kampala | 0.20 (0.00, 0.63) | 1.57 (0.00, 4.08) | 1.37 (0.00, 3.89) |
| South Buganda | 0.56 (0.00, 1.12) | 1.9 (0.41, 3.39) | 1.59 (0.31, 2.87) |
| North Buganda | 8.80 (1.69, 15.92) | 14.17 (4.23, 24.11) | 6.53 (1.64, 11.43) |
| Busoga | 21.21 (11.06, 31.35) | 39.42 (25.60, 53.23) | 24.32 (14.11, 34.52) |
| Bukedi | 3.32 (0.00, 6.80) | 5.34 (0.00, 11.38) | 2.75 (0.00, 6.04) |
| Bugisu | 4.84 (0.00, 9.85) | 10.36 (0.16, 20.56) | 6.88 (0.00, 14.13) |
| Teso | 8.24 (2.37, 14.11) | 20.20 (6.62, 33.78) | 13.65 (2.52, 24.78) |
| Karamoja | 34.3 (24.28, 44.32) | 41.64 (29.36, 53.92) | 19.44 (10.96, 27.93) |
| Lango | 13.35 (6.06, 20.64) | 22.96 (9.32, 36.60) | 12.78 (3.78, 21.79) |
| Acholi | 11.90 (7.48, 16.32) | 28.88 (19.82, 37.93) | 19.69 (12.64, 26.74) |
| West Nile | 21.91 (16.58, 27.25) | 49.99 (39.78, 60.2) | 37.53 (28.29, 46.78) |
| Bunyoro | 9.19 (4.69, 13.69) | 15.21 (7.86, 22.56) | 7.98 (3.53, 12.43) |
| Tooro | 4.73 (1.80, 7.67) | 7.26 (3.2, 11.32) | 3.19 (1.02, 5.36) |
| Ankole | 2.6 (0.00, 6.53) | 2.91 (0.00, 6.45) | 1.02 (0.00, 2.11) |
| Kigezi | 0.28 (0.00, 0.85) | 0.00 | - |
| Refugee settlements | 12.78 (7.74, 17.82) | 32.76 (20.75, 44.77) | 23.37 (13.26, 33.48) |
| By Rurality |  |  |  |
| Urban | 3.28 (0.75, 5.82) | 6.06 (2.48, 9.64) | 3.35 (1.40, 5.30) |
| Rural | 10.85 (8.60, 13.10) | 20.06 (16.42, 23.71) | 11.41 (9.08, 13.75) |

**Table S4. Sample sizes for geospatial covariates.** Missing values were imputed

|  | **n non-missing** | **n missing** |
| --- | --- | --- |
| Altitude | 336 | 4 |
| Aridity index | 304 | 36 |
| Precipitation | 304 | 36 |
| Rainfall | 313 | 27 |
| December temperature | 312 | 28 |
| January temperature | 312 | 28 |
| Land temperature | 312 | 28 |
| Proximity to water | 314 | 26 |
| Enhanced vegetation index | 312 | 28 |
| Diurnal temperature range | 304 | 36 |
| Cattle | 314 | 26 |

**Table S5. Parameter estimates for final Lasso models.** Covariate coding scheme shown in parentheses. "-" indicates that the coefficient was shrunk to 0 by the lasso algorithm.

|  | **Clinical** | **Household** | **Environmental** | **Full** |
| --- | --- | --- | --- | --- |
| Best lambda | 0.02 | 0.011 | 0.03 | 0.022 |
| Intercept | -1.811 | -0.325 | -1.21 | -1.369 |
| Age (<12, 12+ months) | -0.209 |  |  | -0.267 |
| Sex (female vs male) | - |  |  | - |
| LLIN (yes vs no) | - |  |  | - |
| Care seeking for fever in last 2 weeks (yes vs no) | - |  |  | - |
| Antimalarials taken in last 2 weeks (yes vs no) | 0.486 |  |  | 0.485 |
| Antibiotics taken in last 2 weeks (yes vs no) | -0.595 |  |  | -0.391 |
| Antipyretics taken in last 2 weeks (yes vs no) | - |  |  | - |
| Blood taken in last 2 weeks (yes vs no) | - |  |  | - |
| Anemia (Hb < 11 g/dL) | 0.653 |  |  | 0.520 |
| Moderateanemia (Hb < 8 g/dL) | - |  |  | - |
| Mother's age (ordinal) |  | - |  | - |
| Mother's education (ordinal) |  | -0.137 |  | -0.036 |
| Household size (ordinal) |  | - |  | - |
| Urban cluster (yes vs no) |  | -0.617 |  | -0.374 |
| Rural cluster (yes vs no) |  | - |  | - |
| Refugee cluster (yes vs no) |  | 0.206 |  | 0.265 |
| Electricity (yes vs no) |  | - |  | - |
| Owns livestock (yes vs no) |  | - |  | - |
| Drinking water (piped vs not) |  | -0.434 |  | -0.079 |
| Household wealth index (ordinal: 1-5) |  | -0.218 |  | -0.107 |
| IRS in last 12 months (yes vs no) |  | -0.934 |  | -0.529 |
| CHW in community (yes vs no) |  | - |  | - |
| Altitude (2015) |  |  | -0.172 | - |
| Aridity index (2015) |  |  | - | - |
| December temperature (2015) |  |  | - | 0.141 |
| Diurnal temperature range (2015) |  |  | - | - |
| Enhanced vegetation index (2015) |  |  | - | - |
| Land temperature (2015) |  |  | - | - |
| Livestock/cattle (2015) |  |  | - | - |
| January temperature (2015) |  |  | - | - |
| Precipitation (2015) |  |  | - | - |
| Proximity to water (2015) |  |  | - | - |
| Rainfall (2015) |  |  | - | - |
| Region-level microscopy prevalence (2018-2019 MIS) |  |  | 0.372 | 0.310 |

**Supplemental figure legends**

**Figure S1. Flow chart outlining inclusion and exclusion criteria.** Created with BioRender.com.

**Alt text:** Flow chart depicting the number of individuals remaining in the analysis dataset after exclusion criteria applied.

**Figure S2. Observed relationship between region-level malaria prevalence as measured by microscopy (A) and by mRDT (B) among all children vs false-positive mRDT prevalence among microscopy-negative children.** **Plots (C) and (D) display the same data with one outlier (Karamoja) removed.** The sample size for each region is indicated by the point size. The LOESS curve is shown in blue.

**Alt text:** Scatterplots showing the relationship between malaria prevalence as measured by microscopy and mRDT with false-positive mRDT prevalence with smooth LOESS curves overlaid. Point size indicates the sample size.

**Figure S3. Observed relationship between geospatial covariates of interest and false-positive mRDT prevalence among microscopy-negative children by cluster.** The LOESS curve is shown in blue. Missing data is suppressed.

**Alt text:** Scatterplots illustrating the relationship between several geospatial covariates of interest with false-positive mRDT prevalence with smooth LOESS curves overlaid.

**Figure S4. Observed relationship between age in months and the false-positive mRDT prevalence among microscopy-negative children under 5 years.** The sample size for each age is indicated by the point size. The LOESS curve is shown in blue.

**Alt text:** Scatterplot illustrating the relationship between the child's age in months of interest and false-positive mRDT prevalence with a smooth LOESS curve overlaid. Point size indicates the sample size.

**Figure S5. Forest plot of false-positive mRDT prevalences among microscopy-negative children with corresponding 95% confidence intervals for study participants overall and by subgroup of child demographic and clinical characteristics (A) and by subgroup of mother and household characteristics (B).**

**Alt text:** Forest plots displaying prevalence (points) with corresponding 95% confidence intervals (bars), and a dashed line indicating the null value, for all levels of covariates of interest.

**Figure S6. Directional variable importance of retained variables for models developed from environmental candidate predictors, clinical candidate predictors, and household candidate predictors.** The variable importance is the absolute magnitude. The most important variable has importance of 100 or -100. All other variables are ranked relative to the most important. Variable importance less than 0 indicates that the covariate is associated with a decreased probability of a false-positive mRDT. Variable importance greater than 0 indicates that the covariate is associated with an increased probability of a false-positive mRDT.

**Alt text:** Directional bar graph of covariates in the clinical model, environmental model, and household model, with bar magnitude indicating variable importance and bar direction indicating whether the covariate is associated with a decreased or increased probability of a false-positive mRDT.

**References**

1. MIS Overview [Internet]. The DHS Program. [cited 2024 Sep 16]. Available from: https://dhsprogram.com/methodology/survey-types/mis.cfm

2. Uganda National Malaria Control Division (NMCD), Uganda Bureau of Statistics (UBOS), and ICF. Uganda Malaria Indicator Survey 2018-19. Kampala, Uganda, and Rockville, Maryland, USA: NMCD, UBOS, and ICF; 2020.

3. Elkasabi, Mahmoud, Ren, Ruilin, Pullum, Thomas W. Multilevel modeling using DHS surveys: a framework to approximate level-weights. Rockville, Maryland, USA: ICF.; 2020. (DHS Methodological Reports No. 27).

4. Mayala, Benjamin, Fish, Thomas D, Eitelberg, David, Dontamsetti, Trinadh. The Geospatial Covariate Datasets Manual: Second Edition. Rockville, MD, USA: ICF; 2018.

5. Uganda Bureau of Statistics. Lakes and Rivers: Uganda, 2005 [Internet]. 2012. Available from: http://purl.stanford.edu/fh022bz4757

6. The Demographic and Health Surveys Program. ICF International. Boundaries [Internet]. Spatial Data Repository. [cited 2025 Jan 15]. Available from: spatialdata.dhsprogram.com

7. Buuren S van, Groothuis-Oudshoorn K. mice: Multivariate Imputation by Chained Equations in R. Journal of Statistical Software. 2011 Dec 12;45:1–67.

8. Lumley T. Complex Surveys: A Guide to Analysis Using R. John Wiley & Sons; 2011. 329 p.

9. Harel O, Schafer JL. Multiple imputation in two stages. In: Proceedings of federal committee on statistical methodology 2003 conference [Internet]. 2003. Available from: https://www.researchgate.net/profile/Ofer-Harel/publication/253313475_Multiple_Imputation_in_Two_Stages/links/53f5e85a0cf2fceacc6f7bd0/Multiple-Imputation-in-Two-Stages.pdf

10. Du J, Boss J, Han P, Beesley LJ, Kleinsasser M, Goutman SA, et al. Variable Selection with Multiply-Imputed Datasets: Choosing Between Stacked and Grouped Methods. Journal of Computational and Graphical Statistics [Internet]. 2022 Oct 2 [cited 2025 Mar 20]; Available from: https://www.tandfonline.com/doi/abs/10.1080/10618600.2022.2035739

11. Wieczorek J, Guerin C, McMahon T. K-fold cross-validation for complex sample surveys. Stat. 2022;11(1):e454.

12. Cawley GC, Talbot NL. On over-fitting in model selection and subsequent selection bias in performance evaluation. The Journal of Machine Learning Research. 2010;11:2079–107.

13. Lewis MJ, Spiliopoulou A, Goldmann K, Pitzalis C, McKeigue P, Barnes MR. nestedcv: an R package for fast implementation of nested cross-validation with embedded feature selection designed for transcriptomics and high-dimensional data. Bioinformatics Advances. 2023 Jan 1;3(1):vbad048.

14. Kuhn M. Building Predictive Models in R Using the caret Package. Journal of Statistical Software. 2008 Nov 10;28:1–26.

15. Iparragirre A, Lumley T, Barrio I, Arostegui I. Variable selection with LASSO regression for complex survey data. Stat. 2023;12(1):e578.

16. Grau J, Grosse I, Keilwagen J. PRROC: computing and visualizing precision-recall and receiver operating characteristic curves in R. Bioinformatics. 2015 Aug 1;31(15):2595–7.
