# Supplementary figures and images for: False-positive Malaria Rapid Diagnostic Tests are Prevalent Among Children Under 5 Years of Age in Uganda
Source: J Infect Dis. 2025 Nov 28;233(3):e782–90. doi: 10.1093/infdis/jiaf604 (PMC12880570; doi:10.1093/infdis/jiaf604)

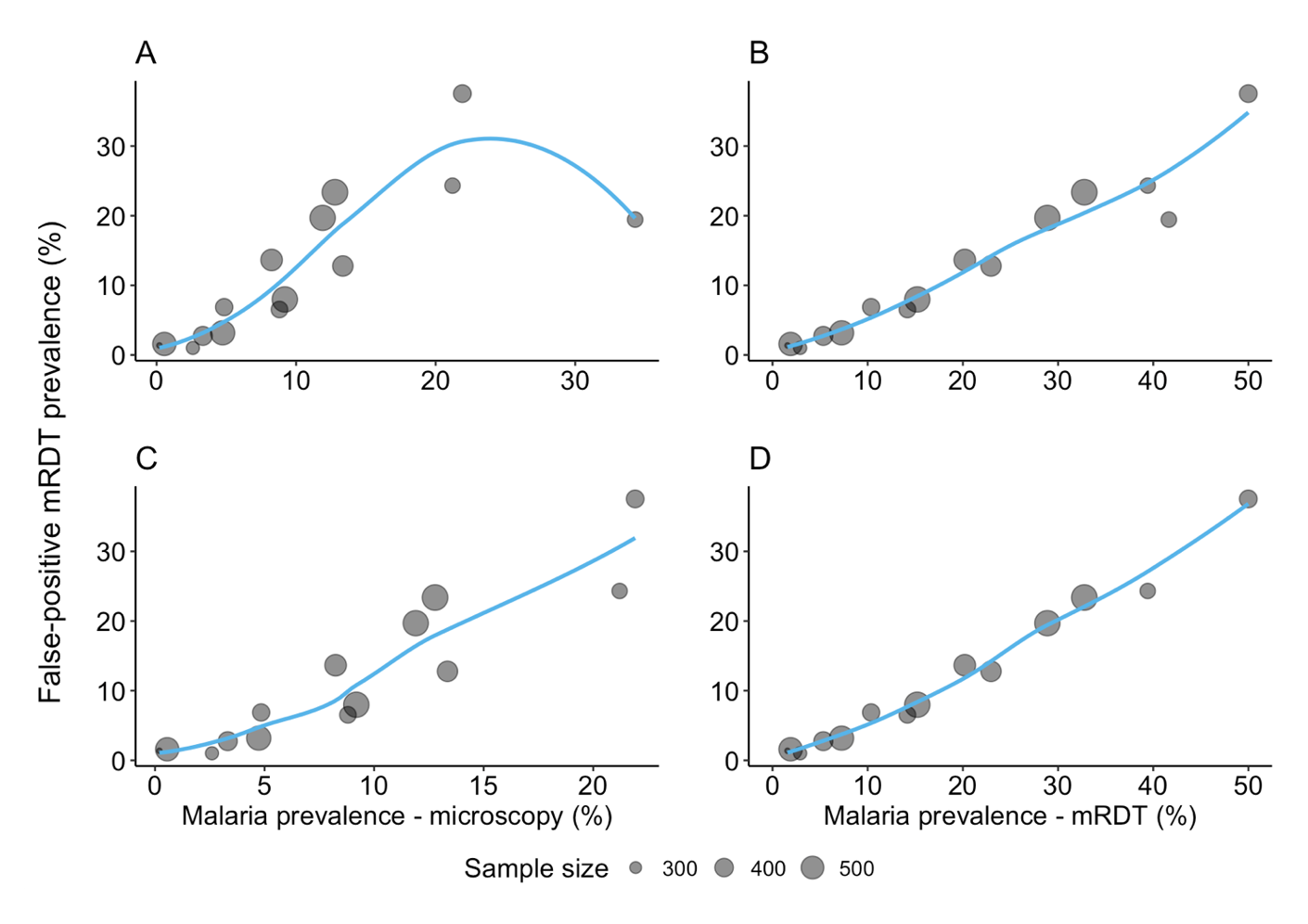

Supplement: jiaf604_Supplementary_Data [file jiaf604_supplementary_data.zip › figS2.tiff]

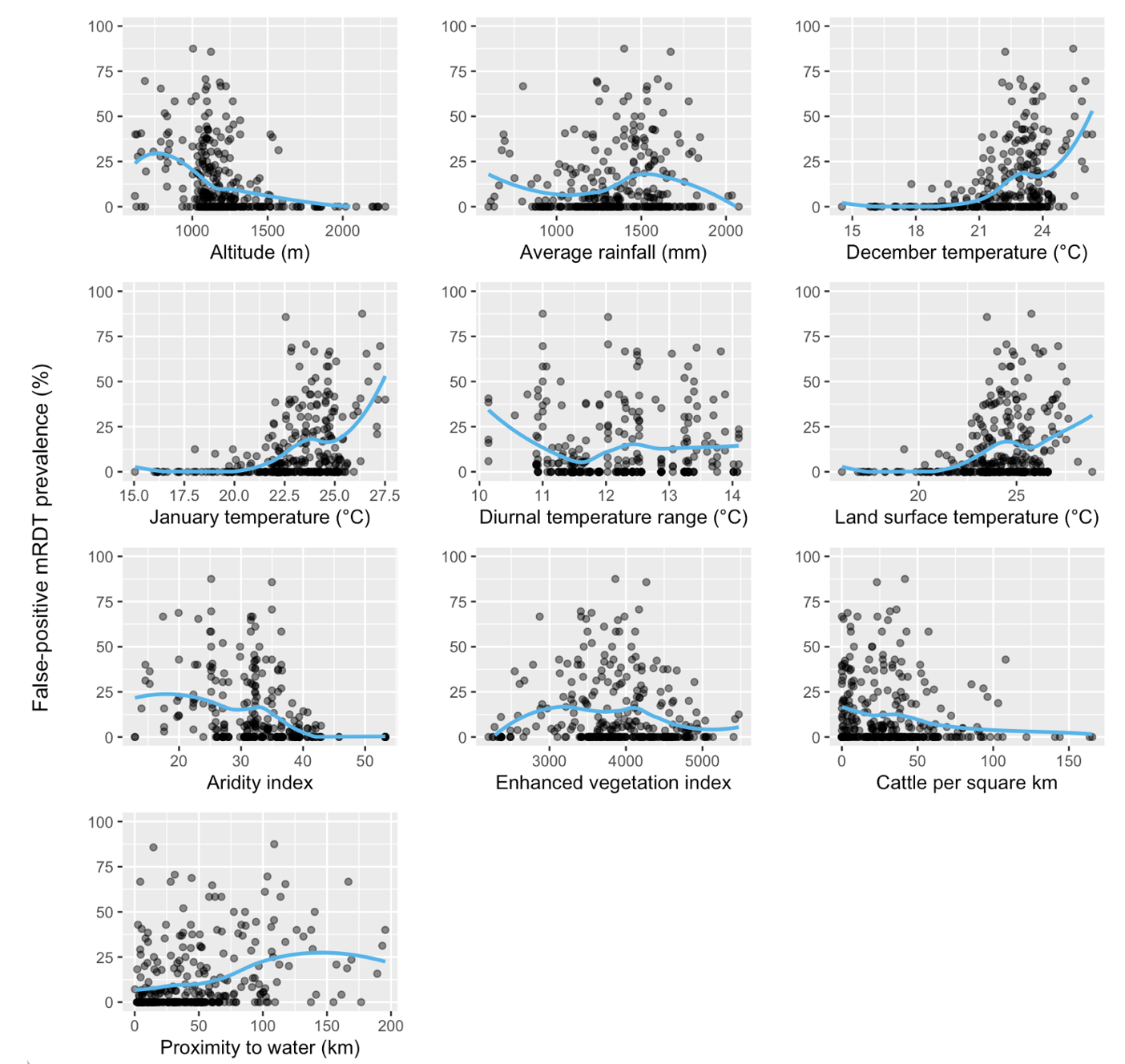

Supplement: jiaf604_Supplementary_Data [file jiaf604_supplementary_data.zip › figS3.tiff]

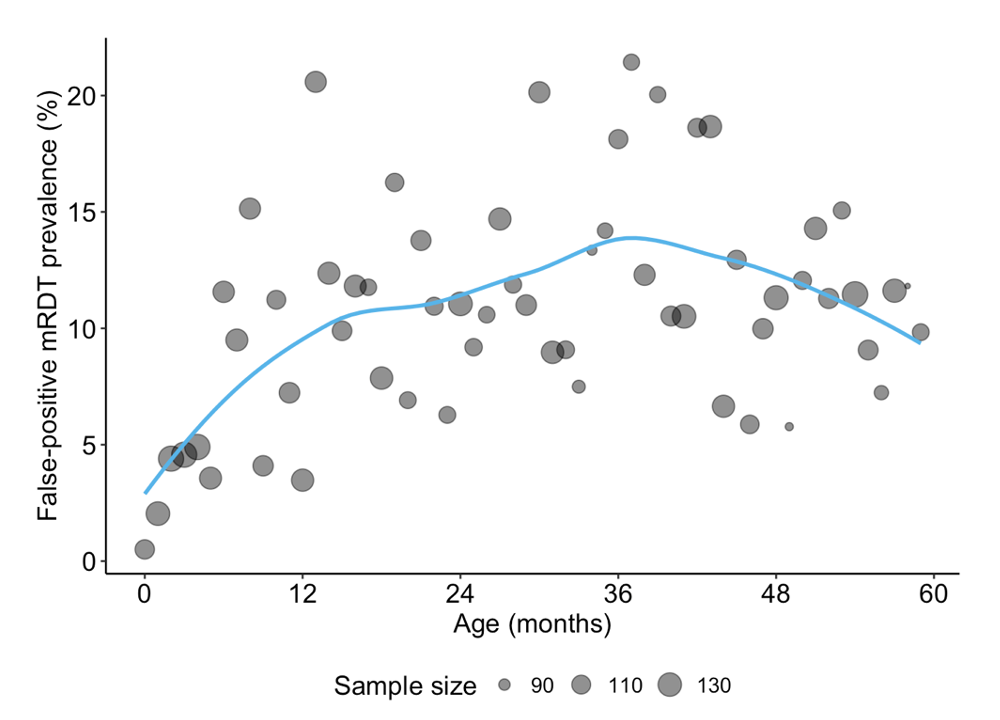

Supplement: jiaf604_Supplementary_Data [file jiaf604_supplementary_data.zip › figS4.tiff]

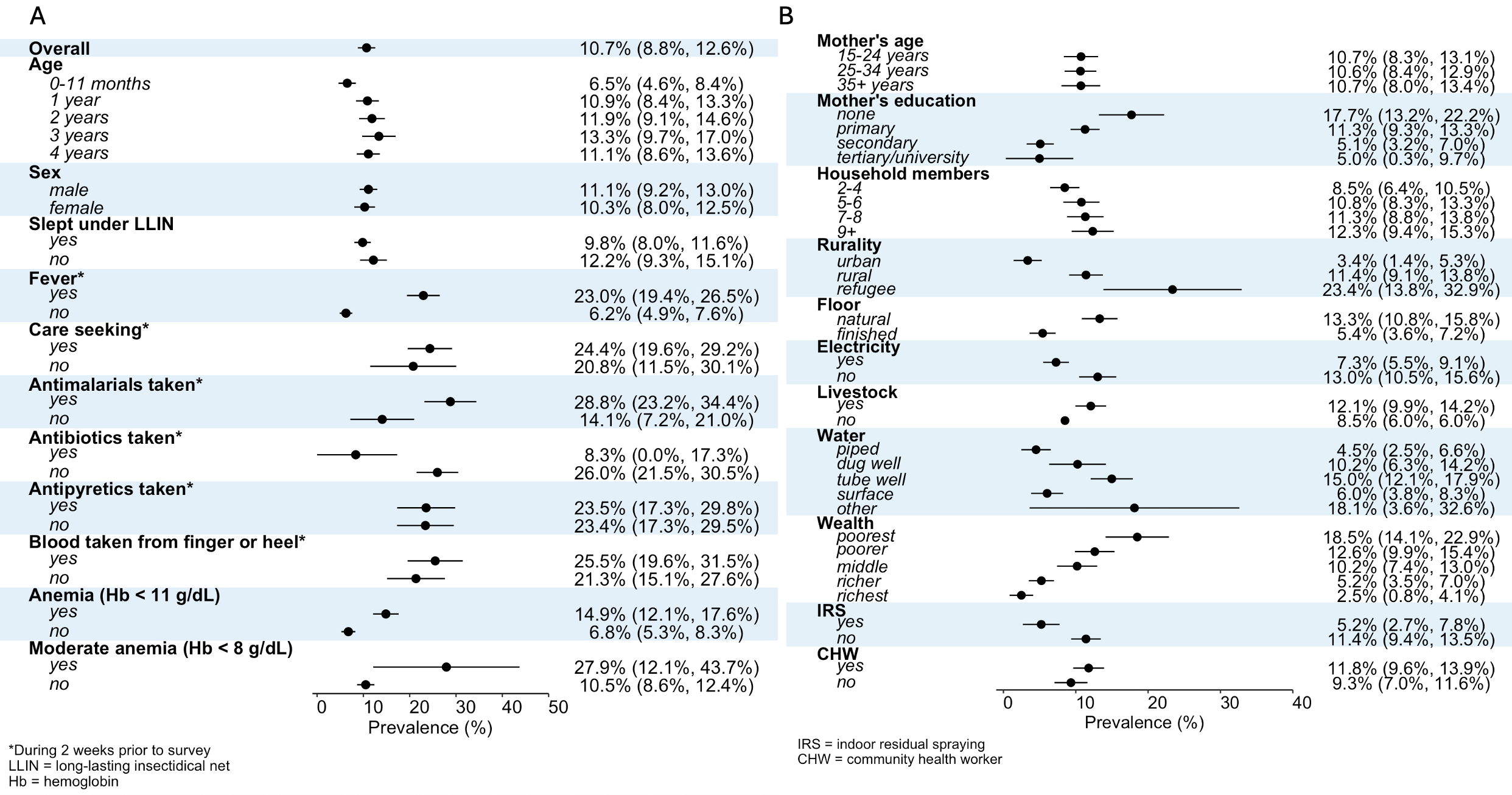

Supplement: jiaf604_Supplementary_Data [file jiaf604_supplementary_data.zip › figS5.tiff]

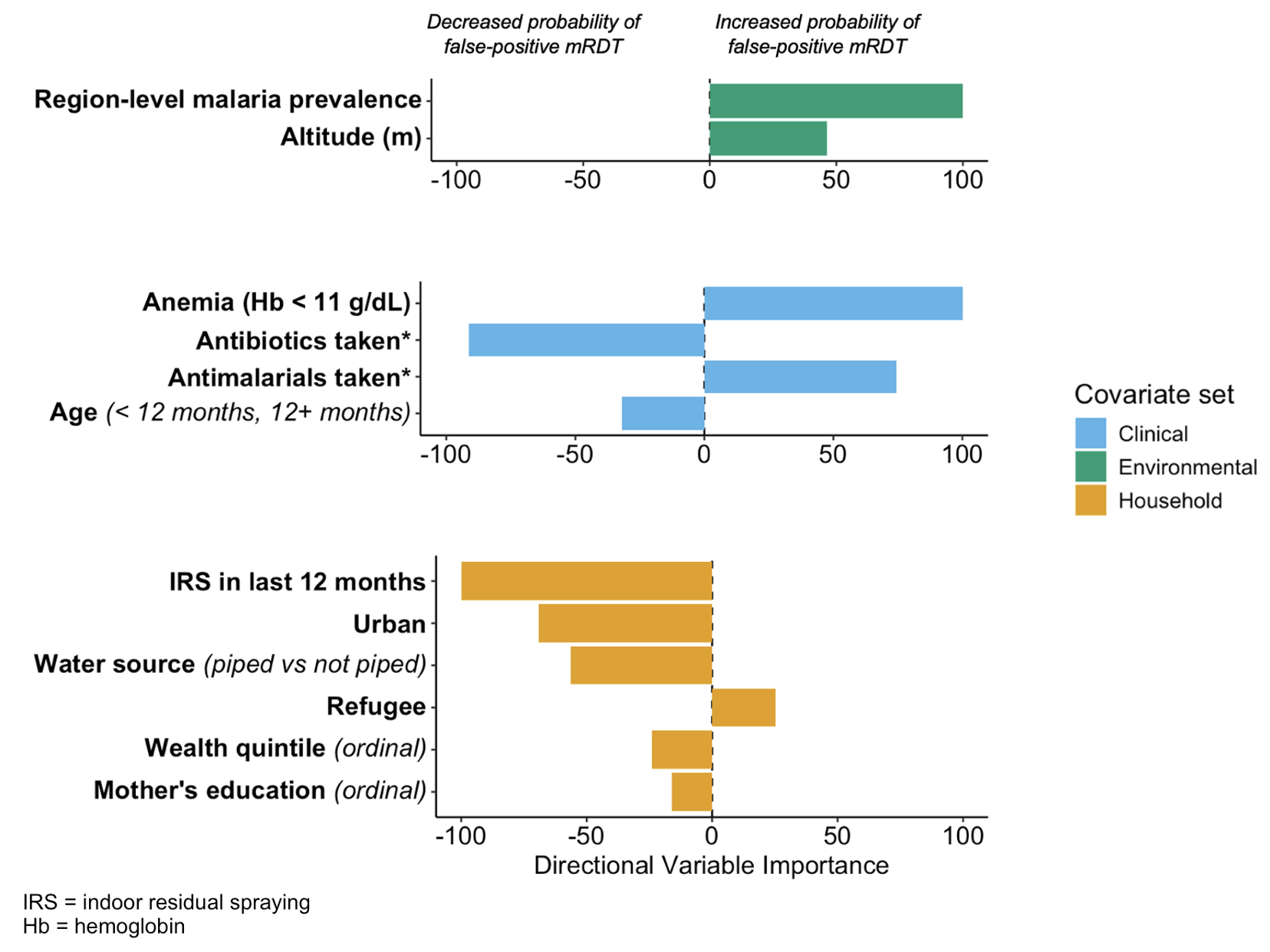

Supplement: jiaf604_Supplementary_Data [file jiaf604_supplementary_data.zip › figS6.tiff]
